# Survival Outcomes and Predictive Factors of Mortality in Feline Epilepsy: A Comprehensive Retrospective Study

**DOI:** 10.3390/ani15111504

**Published:** 2025-05-22

**Authors:** Kreevith Prompinichpong, Nirut Suwanna, Vachira Hunprasit, Amonrat Thongbai, Wutthiwong Theerapan, Naris Thengchaisri, Panpicha Sattasathuchana

**Affiliations:** 1 Kasetsart University Veterinary Teaching Hospital, Bangkhen Campus, Faculty of Veterinary Medicine, Kasetsart University, Bangkok 10900, Thailand; p.kreevith@gmail.com; 2 Department of Companion Animal Clinical Sciences, Faculty of Veterinary Medicine, Kasetsart University, Bangkok 10900, Thailand; nirut.s@ku.th (N.S.); amonrat.np@gmail.com (A.T.); wutthiwong.t@ku.th (W.T.); ajnaris@yahoo.com (N.T.); 3 Department of Veterinary Medicine, Faculty of Veterinary Science, Chulalongkorn University, Bangkok 10330, Thailand; vachira.h@chula.ac.th

**Keywords:** epilepsy, cat, magnetic resonance imaging, seizure, survival

## Abstract

Epilepsy is the most common neurological disorder in cats. However, information on the long-term outcomes and predictive survival factors based on neurological and clinicopathological findings is limited. We aimed to evaluate the clinical manifestations, survival rates, and hazard factors influencing survival in cats with epilepsy by analyzing the medical records of 90 cats diagnosed via brain magnetic resonance imaging (MRI). The cats were divided into the survival and non-survival groups. Univariate and multivariable logistic regression analyses were conducted to identify the significant survival factors. The mortality rates at one and two years were 33.2% and 37.8%, respectively. The median age of cats in the non-survival and survival group were 3 and 1 years, respectively. Age over 7 years (*p* = 0.002), paresis (*p* = 0.001), structural brain lesions (*p* = 0.015), leukocytosis (*p* = 0.001), neutrophilia (*p* = 0.001), hyperproteinemia (*p* = 0.037), hypoalbuminemia (*p* = 0.001), hyperglobulinemia (*p* = 0.003), and an elevated neutrophil-to-lymphocyte ratio (*p* = 0.041), were associated with an increased mortality rate. A multivariable analysis identified several predictors of early mortality in cats with epilepsy, including age over 7 years (*p* = 0.045), presence with paresis (*p* = 0.012), structural brain lesions (*p* = 0.042), leukocytosis (*p* = 0.005), and hypoalbuminemia (*p* = 0.030). Older age and structural brain lesions were shown to be key predictors of mortality and were important for prognosis prediction and management.

## Simple Summary

Cats with epilepsy experience recurring seizures, which are the most common neurological symptoms in cats with brain disorders. In this study, the medical records of 90 cats that had undergone brain magnetic resonance imaging (MRI) was analyzed to identify the factors that had affected their survival over two years. The results showed that the cats older than 7 years and those with limb weakness (paresis), structural brain lesions, and certain blood abnormalities had a lower chance of survival. The cats with structural brain issues lived shorter lives compared to those with non-structural brain lesions. These findings can help veterinarians better predict the prognosis for cats with epilepsy.

## 1. Introduction

Epilepsy is defined as the recurrence of two or more unprovoked seizures at least 24 h apart [1]. Seizures result from abnormal brain activity and are a sign of cortical neuron dysfunction [2]. Although they resemble epilepsy, reactive seizures can occur as a transient response to metabolic or toxic disturbances and disrupt brain function [3]. The International Veterinary Epilepsy Task Force has identified multiple causes of epileptic seizures, including idiopathic epilepsy (IE) and structural epilepsy [4]. IE, which refers to epilepsy without detectable anatomical brain lesions, may have a genetic predisposition [5]. IE is generally diagnosed in dogs and cats aged 6 months to 6 years without metabolic disorder and with no neurological signs between seizures [6]. Structural epilepsy is characterized by epilepsy with evidence of anatomical changes in brain structures [4]. Structural lesions in cats are most commonly multifocal (i.e., found in the cerebrum, brain stem, and/or cerebellum), followed by single lesions in the cerebrum, brain stem, and cerebellum [7]. Idiopathic epilepsy is less common in cats compared to dogs, with seizure manifestations often being more subtle and atypical, requiring a thorough diagnostic work-up due to the lower prevalence of idiopathic epilepsy in cats [8]. A neurological examination is crucial to assess the animal’s mental status, cranial nerve function, motor and sensory responses, and reflexes and to accurately localize the disorder [9]. Magnetic resonance imaging (MRI) has been widely applied in both humans and small animal clinical practices as a non-invasive tool to evaluate the structure of brain diseases [2,10]. The accuracy of *antemortem* brain disease diagnosis has improved with the use of MRI in small animal veterinary practices [11,12,13]. A study showed that inflammation was the most commonly identified structural brain abnormality detected by MRI in cats [7].

Various factors, including uncontrolled seizures, structural abnormalities, and inflammatory brain diseases, have been shown to influence survival rates in humans, dogs, and cats, with uncontrolled seizures in particular increasing the mortality rates of both humans and dogs [14,15]. Dogs and cats with structural epilepsy have shorter survival times compared to those with IE [15,16,17]. The median survival times of dogs from the time of their first seizure due to IE and structural epilepsy were found to be 10.4 and 4.5 years, respectively [15]. The median survival time in a study involving 76 cats with epilepsy of unknown etiology was reported to be 3.2 years [17]. However, limited information is available on the long-term outcomes and key factors influencing survival in epileptic cats based on clinical data, neurological deficits, clinicopathological findings, and MRI results.

The aims of the present study were (1) to evaluate the clinical manifestations (signalments, neurological signs, hematological and biochemical parameters, and the presence of structural brain lesions) in cats with epilepsy, (2) to determine the median survival time of cats with epilepsy, and (3) to identify factors influencing survival in cats with epilepsy that were diagnosed via brain MRI.

## 2. Materials and Methods

### 2.1. Study Period and Location

This study retrospectively evaluated cats with seizure activity that had been diagnosed with epilepsy via brain MRI at the Kasetsart University Veterinary Teaching Hospital, Bangkhen campus, between 1 January 2017, and 30 June 2022.

### 2.2. Case Selection and Data Collection

The inclusion criteria for the present study were cats that had manifested more than one seizure episode and had undergone brain MRI for diagnostic purposes. The enrolled cats were classified into two groups—the survival and non-survival groups—based on their survival from time of their MRI diagnosis. Cats that lived more than two years after the MRI diagnosis were included in the survival group.

The following data were extracted from the medical records: signalment (i.e., age, sex, breed, hair length, body weight, and skull shape), neurological examination findings (i.e., paresis, nystagmus, circling, pupillary light reflex, menace response, gag reflex, and anisocoria), hematological profiles (i.e., hemoglobin, red blood cell count, hematocrit, mean corpuscular volume, mean corpuscular hemoglobin concentration, white blood cell count, neutrophils, lymphocytes, monocytes, eosinophils, basophils, platelets, and neutrophil-to-lymphocyte ratio [NLR]), serum biochemical parameters (i.e., blood urea nitrogen, creatinine, alanine aminotransferase, total protein, albumin, globulin, and albumin-to-globulin ratio), and the presence of structural brain changes on MRI. The hematological and serum biochemical samples were obtained within one week prior to the MRI procedure. The survival time was determined from the time of the MRI diagnosis to either death or the study end date of 29 June 2024. Lost-to-follow-up cases were reviewed via medical records, and owners were contacted by phone if no records were found.

Neurological signs from the medical records included the following abnormalities that are associated with specific brain lesion locations: (1) paresis, assessed by limb mobility and postural deficits; (2) nystagmus, observed through involuntary eye movements; (3) circling, noted as repetitive circular movements; (4) pupillary light reflex, tested by shining a light in each eye and observing pupil constriction; (5) menace response, assessed by observing blink or avoidance when a hand approached the cat’s eyes; (6) gag reflex, induced by stimulating the throat; and (7) anisocoria, evaluated by comparing pupil sizes [8].

A non-structural brain lesion was defined as the absence of detected abnormalities. Structural brain lesions were defined as any abnormalities or deviations from the normal brain structure. Mild changes such as ventricular asymmetry and age-related atrophy, were classified as structural brain lesions. These structural changes were identifiable via various imaging techniques and categorized based on their specific characteristics. These structural changes were assessed via MRI, during which multiple imaging planes were used, including sagittal, dorsal, transverse, T2-weighted, fluid-attenuated inversion recovery (FLAIR), and T1-weighted images, with and without contrast media (gadoterate meglumine; Dotarem^®^, Guerbet LLC, Princeton, NJ, USA). The images were acquired using a 1.5 Tesla MRI system (MAGNETOM ESSENZA, Siemens AG, Erlangen, Germany). Inflammatory brain lesions were diagnosed in the cats that showed T2-weighted hyperintensity or T1-weighted post-contrast enhancement on MRI. All the MRI interpretations were reviewed by Thai board-certified veterinary surgeons who subspecialized in veterinary diagnostic imaging.

### 2.3. Statistical Analysis

The statistical analyses were conducted using the commercially available software packages GraphPad Prism 9.3.1 (GraphPad Software Inc., San Diego, CA, USA) and STATA 12.1 (Stata Corp, College Station, TX, USA). The descriptive statistics were reported as median and range. The Shapiro–Wilk test was used to assess the data normality. Comparisons of the continuous variables (i.e., age, weight, and hematological and biochemical findings) between the cats that did not survive and those that survived were performed using Student’s *t*-test or the Wilcoxon rank sum test, as appropriate.

Fisher’s exact test was chosen over the chi-square test due to low expected frequencies (<5) in some contingency table cells. Fisher’s exact test was conducted to examine the association between survival status and sex, hair length, skull shape, neurological signs (i.e., paresis, nystagmus, circling, pupillary light reflex, menace response, gag reflex, and anisocoria), and structural and inflammatory brain lesions. The Kaplan–Meier estimation method was utilized to estimate the survival function of the variables age (7 years or less/over 7 years) and having a structural brain lesion (yes/no). Death was identified as an event, while data pertaining to loss to follow-up and cats that survived over 7 years were considered right-censored data. The median survival time was calculated based on the Kaplan–Meier method and presented as time with a 95% confidence interval (CI). If the probability of survival did not reach 50%, the 75% (first quartile) probability of survival and the 95% CI were presented. The difference between the survival functions of each category was calculated using the log-rank test. The associations between the time to death and the variables were evaluated by Cox proportional hazard regression. Univariate Cox regression was performed to select the candidate variables. The variables with a *p*-value less than 0.2 based on the univariate Cox regression were selected and included in the multivariable Cox regression analysis [18]. The multivariable Cox regression model was built via a backward stepwise selection based on the likelihood ratio tests to identify independent predictors. The proportional hazards assumption was tested using Schoenfeld residuals [19]. A *p*-value less than 0.05 was considered significant.

## 3. Results

A total of 90 out of 168 cats were enrolled in this study. Seventy-eight cats were excluded due to incomplete medical records, a lack of documented seizure activity, or loss to follow up. Overall, 62.2% of the cats (*n* = 56) survived beyond two years after the diagnostic date, which was based on MRI findings. The one- and two-year mortality rates were 32.2% (*n* = 29) and 37.8% (*n* = 34), respectively. The characteristics of the study population are summarized in Table 1. 

Among the cats, 50 were male (55.6%) and 40 female (44.4%). The predominant breed was Domestic Shorthair (*n* = 53), followed by Persian (*n* = 14), Scottish Fold (*n* = 10), Exotic Shorthair (*n* = 3), American Shorthair (*n* = 2), American Wirehair (*n* = 2), Abyssinian (*n* = 1), American Curl (*n* = 1), British Shorthair (*n* = 1), Chartreux (*n* = 1), Exotic Longhair (*n* = 1), and Sphynx (*n* = 1). The median (range) age of the cats that survived beyond two years (1 [0.3–10.9] years) was significantly lower compared to those that succumbed within this period (3 [0.1–13.8] years, *p* = 0.014). No significant differences in sex (*p* = 0.137), hair length (*p* = 0.788), or skull shape (*p* = 0.814) were found between the cats in the survival and non-survival groups. The MRI findings indicated that 46 cats (51.1%) had structural epilepsy, while 44 cats (48.9%) were classified as having idiopathic epilepsy. An association was detected between the non-surviving group and the presence of neurological abnormalities, including paresis (*p* = 0.001) and anisocoria (*p* = 0.024), as well as the presence of structural brain lesions on MRI (*p* = 0.042).

According to the hematological and biochemical findings, the cats with shorter survival times exhibited elevated mean corpuscular volumes (*p* = 0.001), globulin concentrations (*p* = 0.017), and NLRs (*p* = 0.025; Table 2). Conversely, the non-survival group demonstrated significantly lower concentrations of red blood cells (*p* = 0.001), eosinophils (*p* = 0.020), platelets (*p* = 0.024), and albumin (*p* = 0.033) and lower albumin-to-globulin ratios (*p* = 0.004).

The results of the univariate logistic regression analysis conducted to assess the predictive capability of the clinical features for early mortality are presented in Table 3. The factors associated with increased mortality included age over 7 years (*p* = 0.002), the presence of paresis (*p* = 0.001), the presence of structural brain lesions (*p* = 0.015), leukocytosis (*p* = 0.001), neutrophilia (*p* = 0.001), hyperproteinemia (*p* = 0.037), hyperglobulinemia (*p* = 0.003), hypoalbuminemia (*p* = 0.001), and an elevated NLR (*p* = 0.041).

The subsequent multivariable analysis showed significant associations for the cats age over 7 years (hazard ratio 2.21, 95% CI 1.02–4.81, *p* = 0.045), the presence of paresis (hazard ratio 2.61, 95% CI 1.23–5.54, *p* = 0.012), the presence of structural brain lesions (hazard ratio 2.73, 95% CI 1.04–7.18, *p* = 0.042), leukocytosis (hazard ratio 3.16, 95% CI 1.42–7.06, *p* = 0.005), and hypoalbuminemia (hazard ratio 6.98, 95% CI 1.20–40.44, *p* = 0.030; Table 4). Notably, the highest risk of early mortality was associated with the presence of hypoalbuminemia.

**Figure 1:**
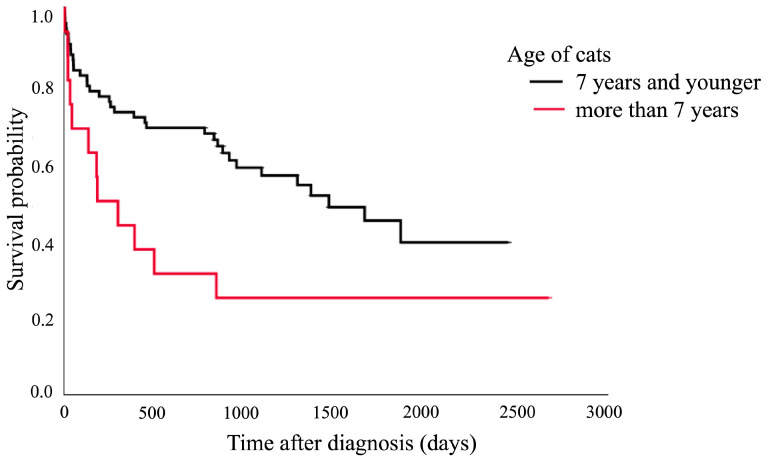
Kaplan–Meier survival curve showing the median survival of the cats aged over 7 years (182 days, 95% CI: 0–413.3 days) and those aged 7 years and younger (1460 days, 95% CI: 804.0–2115.8 days; *p* = 0.020).

**Figure 2:**
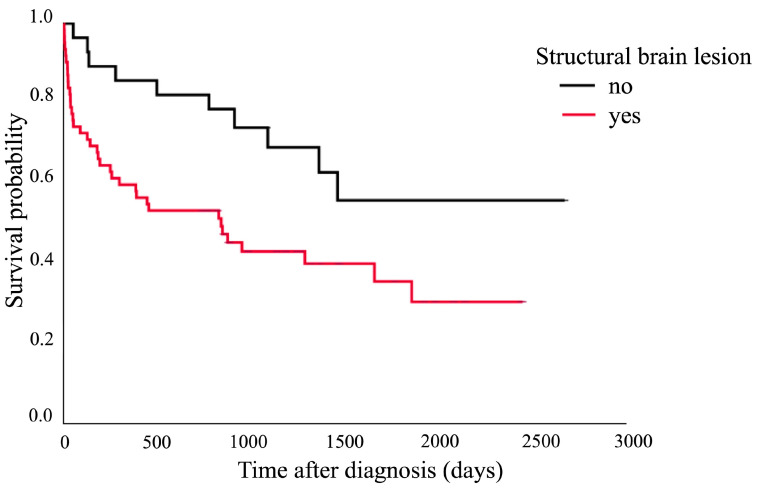
Kaplan–Meier survival curve showing the 75% probability of survival of the cats with structural brain lesions (51 days, 95% CI: 0–152.3 days) and those with non-structural brain lesions (910 days, 95% CI: 152.7–1667.4 days; *p* = 0.016).

The Kaplan–Meier survival curve showed that cats aged over 7 years had a shorter median survival time (182 days, 95% CI: 0–413.3 days) compared to those aged 7 years and younger (1460 days, 95% CI: 804.0–2115.8 days). The comparison between these two groups showed a significant difference (*p* = 0.020), as presented in Figure 1. Figure 2 shows that the cats with structural brain lesions had a shorter survival time, with a 75% probability of survival (51 days, 95% CI: 0–152.3 days), compared to those with non-structural brain lesions (910 days, 95% CI: 152.7–1667.4 days; *p* = 0.016).

## 4. Discussion

The medical records of 90 epileptic cats were reviewed retrospectively in this study, and the median survival time was found to be 720 days. Our findings showed that the long-term survivors were younger than the non-survivors. Paresis, anisocoria, and structural brain lesions were associated with shorter survival, as were elevated mean corpuscular volumes and globulin levels and low red blood cell, eosinophil, platelet, and albumin levels. The factors influencing early mortality, which were determined via the univariate and multivariable analyses, were age over 7 years, paresis, structural brain lesions, leukocytosis, and hypoalbuminemia.

The age of the cat significantly influenced survival, with the younger cats having longer median survival times (i.e., beyond two years) than those in the non-survival group. This could be because younger animals generally have a better prognosis with epilepsy, likely due to their higher recovery capacity and lower age-related decline [20]. The older cats, particularly those over 7 years of age, showed increased mortality, possibly due to the cumulative effects of aging on brain health and seizure-related damage [21].

Neurological signs, including paresis and anisocoria, were linked to shorter survival. Paresis is likely associated with brainstem dysfunction, as the brainstem controls the rubrospinal and reticulospinal tracts, which are vital for gait generation [22]. Similarly, anisocoria may result from brainstem lesions that affect the cranial nerves responsible for pupil response [23]. The cats with paresis or anisocoria in the present study had significantly lower chances of survival. These findings suggested more severe underlying neurological conditions, likely related to structural brain damage or dysfunction—particularly that involving the brainstem, which can impair vital brain functions [24]. This was consistent with the results of a prior study, in which severe neurological deficits were found to predict poor outcomes in dogs with epilepsy [25].

Structural brain lesions also played a key role in survival, with cats having shorter survival times if lesions were present. This finding was consistent with reports from other studies on canine and feline epilepsy, where structural lesions often correlated with a poorer prognosis [26]. MRI findings have suggested that cerebral lesions may contribute to brain dysfunction and the progression of epileptic activity, which ultimately leads to shorter survival times [27]. The most identified structural brain lesion from MRI in a previous study was inflammatory brain lesions, followed by tumors that occupied brain tissues [7]. This emphasizes the importance of imaging in diagnosing and managing epilepsy in cats. The false negative in diagnosing IE in cats may occur due to limitations in MRI resolution. The location of the lesion or microscopic changes may not be visible on conventional 1.5-Tesla MRI [28]. As the present study used a 1.5-Tesla MRI, which is commonly used in both human and veterinary medicine, clinicians should consider the possibility of undetected lesions. Follow-up MRI may be necessary in cats with severe disease progression.

Hematological and biochemical parameters were also linked to survival in this study. The cats with shorter survival times had higher mean corpuscular volumes, globulin levels, and NLRs, along with lower concentrations of red blood cells, eosinophils, platelets, and albumin and lower albumin-to-globulin ratios. These findings may have limited clinical relevance due to minor variations among the cats in the survival group. Furthermore, selection bias may influence the results, as cats that undergo MRI will likely have blood test results within normal reference intervals to ensure anesthetic safety. This criterion may have excluded more severely affected individuals and thus limited the generalizability of the findings. However, in certain cases, cats with hematological and biochemical abnormal test results were included when the diagnostic benefit of MRI was considered to outweigh the anesthetic risk. As a result, some cats with abnormal hematologic or biochemical findings were part of the study cohort. Interestingly, our results indicate that such abnormalities may be associated with increased risk of mortality.

Several hazard factors were found to contribute to reduced survival. Cats over the age of 7 years, the presence of paresis and structural brain lesions, leukocytosis, neutrophilia, hyperproteinemia, hyperglobulinemia, hypoalbuminemia, and an elevated NLR were factors that contributed to a decreased survival time. A cerebrospinal fluid (CSF) analysis in the cats has revealed that abnormalities linked to neuroinflammatory diseases [29]. The CSF analysis results were not evaluated in the present study, as the procedure could not be performed in some cases due to the risk of brain herniation or clinical instability, in order to avoid worsening the cat’s condition.

The present study showed that an NLR > 4 increased the likelihood of early mortality. This finding is in accordance with that from previous research in which cats with an NLR > 4.5 were reported to be at risked of mortalities in cases with cardiac disease [30]. In dogs, elevated NLR has been evaluated in dogs with epilepsy and identified as an important marker for all types of epilepsy, with abnormal NLR values serving as an initial tool to screen for neuroinflammation [31]. An abnormal NLR has been reported to distinguish between meningoencephalitis of unknown etiology and other brain diseases in dogs with epilepsy [32]. Additionally, the NLR could serve as a biomarker for neuroinflammation in epilepsy in humans [33]. These findings suggested that the NLR may serve as a valuable biomarker for neuroinflammation and a potential tool for diagnosis and treatment in feline with epilepsy. However, the NLR cutoffs have not been previously evaluated and validated for cats with neuroinflammation. Further investigations into the diagnosis or prognostic cutoff of the NLR for cats with epilepsy should be undertaken.

In dogs with meningoencephalitis of an unknown etiology, mild, non-clinically relevant changes have been seen in hematology and biochemistry [34]. However, tickborne encephalitis has shown more significant hematologic and biochemical abnormalities [35]. Few studies have explored blood changes in cats with neuroinflammatory diseases. Blood values showing hyperproteinemia, hyperglobulinemia, and hypoalbuminemia, which resulted in a low albumin-to-globulin ratio, suggested feline infectious peritonitis in some cases. Due to the retrospective nature of the current study, feline infectious peritonitis cannot be ruled out. The multivariable analyses revealed that age over 7 years, paresis, structural brain lesions, leukocytosis, and hypoalbuminemia were significant predictors of mortality. Hypoalbuminemia showed the highest hazard ratio but did not reach clinical significance due to possible selection bias in the pre-anesthesia procedure. Among these parameters, age over 7 years and paresis may be more clinically relevant markers for poor outcomes due to their obvious clinical presentations.

Investigations of feline seizures begin by establishing the nature of the episodes based on differential diagnoses, followed by basic hematology and biochemistry tests before considering specific tests, such as bile acid stimulation and infectious disease panels [8]. IE encompasses cases with no identifiable cause or structural disease, including genetic, suspected genetic, or unknown origins. Genetic epilepsy is confirmed through genetic testing, while suspected genetic epilepsy is based on high breed prevalence (>2%) or familial clustering. Unlike canine epilepsy, which has well-established breed predispositions, documenting inheritance patterns in cats is more challenging, especially since most pet cats are of a mixed breed, rescued, or adopted with unknown family lines. Feline epilepsy often arises from a mixed genetic background [36], and some families with spontaneous seizures have shown an autosomal recessive inheritance pattern, although other inheritance patterns cannot be ruled out [37]. Genetic information may prove useful in future studies aimed at better predicting the prognosis of feline IE outcomes.

## 5. Limitations and Recommendations

The present study had several limitations, including its retrospective design; reliance on medical records; potential bias from incomplete or inaccurate data; and the lack of evaluation of anti-epileptic drug response, underlying cause, and postmortem brain histopathological evaluation. Additionally, the use of baseline-only data provides a static analytical framework, limiting the ability to identify clinical changes overtime that may contribute to survival. The single-center approach limited its generalizability, and MRI selection bias based on pre-anesthesia blood values could affect representativeness of the samples. The choice of anti-epileptic drug varied and was based on the neurologist’s judgement, and anti-seizure responses were not evaluated; this could introduce variability in survival predictions. Furthermore, the underlying causes of epilepsy, such as hypoalbuminemia and hyperglobulinemia, were not explored. Postmortem evaluations were not included due to feasibility and owner compliance. Future research should involve larger cohorts in multicenter, prospective studies to explore treatment strategies, prognostic biomarkers, and long-term survival outcomes.

## 6. Conclusions

Older age, neurological deficits (paresis and anisocoria), and structural brain lesions were linked to poorer prognosis and shorter survival in the cats with epilepsy in this study. The multivariable analysis showed that age over 7 years, paresis, structural brain lesions, leukocytosis, and hypoalbuminemia predicted mortality, with hypoalbuminemia being the most significant risk factor. However, its relevance may be limited by potential selection bias due to the pre-anesthetic procedure. Overall, age over 7 years and structural brain lesions were the key predictors of early mortality, which emphasizes the importance of clinicopathological diagnosis and imaging in managing feline epilepsy and improving prognosis. It is therefore important to closely monitor older cats with epilepsy, particularly those showing neurological deficits or structural brain lesions. Early identification and thorough diagnostic investigations, which include imaging and clinicopathological evaluations, can help guide treatment decisions and improve prognoses in cats with epilepsy.

## Figures and Tables

**Table 1:** Characteristics of the cats with epilepsy.

	Values	Non-Survival	Survival	*p*-Value
*N*; %	90; 100%	47; 52.2%	43; 47.8%	
Age (years; median [range])	2 (0.1–13.8)	3 (0.1–13.8)	1 (0.3–10.9)	0.014
Sex (*n*; %)				
Male	50; 55.6%	30; 33.3%	20; 22.2%	0.137
Female	40; 44.4%	17; 18.9%	23; 25.6%	
Hair length (*n*; %)				
Short-haired	74; 82.2%	38; 42.2%	36; 40%	0.788
Long-haired	16; 17.8%	9; 10%	7; 7.8%	
Weight (mean ± SD)	3.6 ± 1.3	3.5 ± 1.3	3.8 ± 1.3	0.336
Skull shape (*n*; %)				
Brachycephalic	25; 27.8%	14; 15.6%	11; 12.2%	0.814
Non-brachycephalic	65; 72.2%	33; 36.7%	32; 35.6%	
Neurological signs (*n*; %)				
Paresis	17; 18.7%	15; 16.7%	2; 2.2%	0.001
Non-paresis	73; 81.1%	32; 35.6%	41; 45.6%	
Isocoria	68; 75.6%	21; 61.8%	47; 83.9%	0.024
Anisocoria	22; 24.4%	13; 38.2%	9; 16.1%	
Structural lesions (*n*; %)				
Structural	46; 51.1	37; 41.1%	25; 27.8%	0.042
Non-structural	44; 48.9%	10; 11.1%	18; 20.0%	
Inflammatory lesions (*n*; %)				
Inflammatory	39; 43.3%	22; 24.4%	17; 18.9%	0.528
Non-inflammatory	51; 56.7%	25; 27.8%	26; 28.9%	

**Table 2:** Hematological and biochemical profiles of the cats with epilepsy, shown as medians (ranges).

Parameters	Value	Non-Survival	Survival	*p*-Value
Hemoglobin (g/dL)	12.1 (7.4–16.1)	11.8 (7.7–15)	12.3 (7.4–16.1)	0.142
Red blood cells (10^6^ cells/µL)	8.1 (4.7–12.1)	7.61 (4.7–11)	8.73 (5.7–12.1)	0.001
Hematocrit (%)	34.6 (21.9–49.7)	33.7 (22.2–49.7)	35.1 (21.9–49.7)	0.380
Mean corpuscular volume (fL)	42.8 (32.5–62.2)	45.7 (32.5–62.2)	40.6 (32.8–51.2)	0.001
Mean corpuscular hemoglobin concentration (g/dL)	34.5 (27.5–40.9)	34.3 (27.5–40.9)	34.8 (31–38.3)	0.311
White blood cells (10^3^ cells/µL)	11.7 (4.2–36.4)	11.5 (4.8–36.4)	12.4 (4.2–26.5)	0.922
Neutrophils (10^3^ cells/µL)	8.3 (0.6–29.5)	9.3 (0.6–29.5)	8.0 (1.7–20.1)	0.171
Lymphocytes (10^3^ cells/µL)	2.0 (0.2–19.8)	1.8 (0.2–19.8)	2.5 (0.7–11.7)	0.082
Monocytes (10^3^ cells/µL)	0.2 (0–2.9)	0.2 (0–2.9)	0.2 (0–1.6)	0.121
Eosinophils (10^3^ cells/µL)	0.3 (0–3.3)	0.2 (0–3.3)	0.4 (0–2.2)	0.020
Basophils (10^3^ cells/µL)	0.0 (0–0.1)	0.0 (0–0.1)	0.0 (0–0.1)	0.278
Platelets (10^3^ cells/µL)	248.5 (10–595)	226 (10–438)	266 (22.7–595)	0.024
Blood urea nitrogen (mg/dL)	22 (6–45)	22 (10–43)	22 (6–45)	0.680
Creatinine (mg/dL)	1.3 (0.4–2.6)	1.3 (0.4–2.6)	1.3 (0.5–2.5)	0.375
Alanine amino transferase (U/L)	59.5 (17–730)	60 (17–266)	59 (21–730)	0.966
Total protein (g/dL)	7 (5.3–9.5)	7 (5.3–9.5)	7 (5.6–8.8)	0.373
Albumin (g/dL)	3.4 (2.4–4.3)	3.4 (2.4–4.1)	3.5 (2.6–4.3)	0.033
Globulin (g/dL)	3.6 (2.4–5.9)	3.7 (2.4–5.9)	3.4 (5.6–5.0)	0.017
Albumin–globulin ratio	0.94 (0.5–1.5)	0.9 (0.5–1.3)	1 (0.6–1.5)	0.004
Neutrophil–lymphocyte ratio	3.7 (0.1–48.5)	4.6 (0.2–48.5)	3.2 (0.3–18.8)	0.025

**Table 3:** Univariate analysis results showing the predictors of mortality in the cats with epilepsy.

Parameters	Number of Cats That Had an Event	Hazard Ratio (95% CI)	*p*-Value
Age over 7 years	12	3.08 (1.49–6.34)	0.002
Male gender	30	1.45 (0.72–2.89)	0.296
Brachycephalic breed	14	0.82 (0.37–1.80)	0.614
Paresis	15	3.36 (1.66–6.78)	0.001
Anisocoria	13	2.36 (1.18–4.74)	0.015
Structural brain lesions	37	3.26 (1.26–8.42)	0.015
Anemia (hematocrit < 25%)	2	0.49 (0.07–3.57)	0.480
Leukocytosis (>19.0 × 10^3^ cells/µL)	10	3.55 (1.68–7.52)	0.001
Neutrophilia (>12.5 × 10^3^ cells/µL)	16	3.06 (1.55–6.08)	0.001
Lymphocytosis (>7.0 × 10^3^ cells/µL)	3	1.02 (0.25–4.28)	0.974
Monocytosis (>0.9 × 10^3^ cells/µL)	3	0.83 (0.25–2.72)	0.759
Thrombocytopenia (<200 × 10^9^ cells/µL)	12	1.92 (0.93–3.93)	0.076
Elevated blood urea nitrogen (>34 U/L)	6	2.37 (0.98–5.76)	0.056
Elevated creatinine (>2.2 mg/dL)	1	1.76 (0.24–12.90)	0.577
Elevated alanine aminotransferase (>150 U/L)	3	1.16 (0.36–3.81)	0.800
Hyperproteinemia (>7.8 mg/dL)	9	2.32 (1.05–5.15)	0.037
Hypoalbuminemia (<2.6 mg/dL)	2	11.55 (2.56–51.97)	0.001
Hyperglobulinemia (>5.1 mg/dL)	12	3.00 (1.45–6.17)	0.003
Elevated neutrophil–lymphocyte ratio (>4)	34	2.05 (1.03–4.10)	0.041

**Table 4:** Multivariable analysis results showing the predictors of mortality in cats with epilepsy.

Parameters	Hazard Ratio (95%CI)	*p*-Value
Age over 7 years	2.21 (1.02–4.81)	0.045
Paresis	2.61 (1.23–5.54)	0.012
Structural brain lesions	2.73 (1.04–7.18)	0.042
Leukocytosis (>19.0 × 10^3^ cells/µL)	3.16 (1.42–7.06)	0.005
Hypoalbuminemia	6.98 (1.20–40.44)	0.030

## Data Availability

The data presented in this study are available within this article. The raw data supporting this study are available from the corresponding author upon reasonable request.

## Abbreviations

The following abbreviations are used in this manuscript:

CI	confidence interval
CSF	cerebrospinal fluid
IE	idiopathic epilepsy
MRI	magnetic resonance imaging
NLR	neutrophil-to-lymphocyte ratio
